# Facilitating point-of-care detection/suspicion of early TB disease to enable early treatment access, while awaiting more definitive microbiologic diagnosis

**DOI:** 10.7448/IAS.19.1.21007

**Published:** 2016-03-24

**Authors:** Diana B Dickinson

**Affiliations:** Independence Surgery, Gaborone, Botswana

Tuberculosis (TB) was the scourge of the past few centuries among European populations, causing up to 40% mortality within the first year of diagnosis [1]. Effective chemotherapy dramatically improved prognosis [1,2]. However, the early 1980s [2] heralded another resurgence of tuberculosis, this time with even higher mortality, reaching 55% in some Sub-Saharan countries [1] due to the explosion of the HIV epidemic and its accompanying immunosuppression [3]. This rapid increase in morbidity and mortality of tuberculosis started levelling off from 2004 [2] due to increasing access to combination antiretroviral therapy (cART) for HIV-positive people in Africa.

Nevertheless, TB was still responsible for 1,200,000 deaths worldwide in 2014 [4], 400,000 in those with HIV/TB co-infection [4]. There were 9.6 million new cases of TB in 2014, 28% in Africa alone (home to only 7% of the world's population!) [2,4]. In Southern Africa, it is estimated that TB/HIV co-infection is greater than 60% [3], and nine of these Southern African countries, including Botswana, are responsible for nearly 50% of this global disease burden. In fact this translates to 74% of coinfected cases residing in the African region [4].

Unfortunately, as immunodeficiency progresses during the course of HIV infection, TB diagnostic challenges increase and standard TB diagnostics become less useful [5–11] unless the test is positive. HIV/TB co-infection alters clinical presentation and course of TB, increasing dissemination [3] and early mortality [12–15], and reducing sensitivity of sputum examination and chest X-ray [16]. In practise, severely immunosuppressed patients more commonly have sputum smear-negative (SSN), ranging from 25 to 61% in Sub-Saharan Africa [4,17–19]. These SSN patients are twice as likely to die [1,8,19], probably because of diagnostic uncertainty with the resultant delay in administering the appropriate therapy. In addition, increasing immunosuppression with advancing HIV results in more extra-pulmonary TB (EPTB), which is more difficult to prove bacteriologically but is all the more common in our setting than pulmonary TB, and frequently missed. Several autopsy studies suggest that fewer than *half* of the TB cases are diagnosed before death [20–22], which highlights the impact that *timely diagnosis* of TB may have on early mortality and morbidity of co-infected patients initiating cART. Nearly 100% of these are EPTB. Strang [23] puts it more forcefully: Early TB diagnosis saves lives!

Importantly, TB symptoms are notoriously vague, especially in EPTB. In South Africa, expanding their national diagnostic algorithm to two weeks of cough, fever, and night sweats with unexplained weight loss of more than 1.5 kg in a month, increased their diagnostic sensitivity to 96%, but of course reducing specificity.

*Confirmed* TB is proven microbiologically by culture or molecular tests. *Probable* TB is suggested by microscopic and histological means. But in most cohorts in Africa, even with stringent testing, 60% of all cases are only *possible*, suggested by chest X-ray or clinical grounds [15].

Definitive diagnosis in low-resourced countries is challenging, and often impossible [15]. Initially seeing a chronically sick patient, and suspecting disseminated TB, the first and fastest microbiological test for probable TB confirmation would be sputum microscopy for acid-fast-bacilli (AFB), (fast, cheap, easy, but insensitive [2,15,17,18]). This has a turnaround time (TAT) of 48 hours in the South African TB Programme (personal communication). More sensitive (still only 60–70% sensitive in immunosuppressed patients) but giving definitive results, a Cepheid GeneXpert nucleic acid amplification test (NAAT), a bio-marker on sputum or other tissue, also takes 48 hours and has revolutionized early diagnosis of TB. The new GeneXpert ultra [4] may improve this sensitivity. It is also more expensive, as is a Line Probe Assay.

Chest X-rays can be done within 24 hours, and can indicate high possibility of TB.

Tissue biopsy logistically must take longer but also may only indicate a possible diagnosis of TB. Blood and secretion cultures give definitive diagnosis but are often unavailable, expensive, complicated, and even slower (although Bactec Mycobacterium Growth Indicator Tube (MGIT) System has reduced earliest TAT significantly to even as little as five days – not counting time taken to reach the health provider). Solid cultures (Lowenstein–Jensen) may take up to six weeks for a result, by which time the patient may have died.

But these are not point-of-care technologies! Point-of-care implies an aid to the clinical examination that gives immediate feedback, without specimens being sent to a laboratory or patients themselves to a special unit, thus pre-empting possible logistical delays/mistakes before the results are known. This usually involves traditional membrane-based test strips, for example, finger-prick blood or urine tests, with portable ultrasonography being included.

It is essential to be able to increase the yield of possible TB diagnosis in these highly immunocompromised, at-risk patients. At the point-of-care, empiric diagnosis and treatment using clinical acumen is often the only resource available. Could echocardiography at the bedside assist in these critically ill patients? Ultrasound machines are often available even at the remotest outposts. In fact, portable, small, phone-sized ultrasounds can be taken on outreach trips to isolated clinics. In particular, detection of TB pericarditis using echocardiography has proved to be feasible in Africa [24,25], affecting outcomes favourably and allowing earlier hospital discharges. This then could prove useful at the bedside, with the typical appearances of thickened heavily frayed pericardium with a thick fibrinous pericardial effusion providing compelling indications and suggesting an immediate high possibility of disseminated TB, enabling early initiation of Anti-Tuberculosis-Therapy (ATT). Given a background of high TB burden, most patients (but not all) with this result will have TB, and a trial of treatment will be vindicated. These patients will be diagnosed in addition to those with more easily diagnosed pulmonary TB. Of course, all appropriate confirmatory tests should be done; all the possible sputum, blood, and molecular tests would be instituted simultaneously with the initiation of ATT. Then one can wait for these confirmatory tests to return, (or not, while hopefully the patient is at least starting to improve clinically).

Ultrasonography could be easily implemented in most developing countries [24], where the burden of TB/HIV disease is high, without complicated requirements for extra, sophisticated equipment or extensive training. Many clinicians have obstetric echocardiograms already on site. Specialist radiologists, although desirable, are unnecessary. Motivated paramedical personnel could easily be trained for these tasks. In addition, extra materials such as reagents are unnecessary.

In our clinic setting in Botswana, routine ultrasounds by family practitioners on newly presenting, profoundly immunosuppressed patients reveal early pericarditis with frayed thickened pericardium and fibrinous effusions very frequently, prompting initiation of ATT and cART- that is combination anti-retroviral-therap with very good outcomes. A greater index of suspicion is necessary, and perhaps access to an ultrasound of any sort may be another point-of-care adjunct facilitating early detection of TB, improving morbidity and mortality rates in this at-risk population.
